# Proportional dose of rapid-onset opioid in breakthrough cancer pain management: An open-label, multicenter study

**DOI:** 10.1097/MD.0000000000011593

**Published:** 2018-07-27

**Authors:** Tsung-Yu Yen, Jeng-Fong Chiou, Wei-Yong Chiang, Wen-Hao Su, Ming-Yuan Huang, Ming-Hung Hu, Shen-Chi Wu, Yuen-Liang Lai

**Affiliations:** a Department of Radiation Oncology; b Hospice and Palliative Care Center, MacKay Memorial Hospital; c Department of Radiology, School of Medicine, College of Medicine, Taipei Medical University; d Department of Radiation Oncology, Taipei Medical University Hospital; e Taipei Cancer Center, Taipei Medical University; f Cancer Center, Taipei Medical University Hospital, Taipei; g Department of Palliative Medicine; h Division of Hematology and Oncology, Department of Medicine, Cardinal Tien Hospital; i School of Medicine, Fu Jen Catholic University, New Taipei; j Department of Hospice and Palliative Care, Taipei; k MacKay Medical College, New Taipei, Taiwan.

**Keywords:** breakthrough cancer pain, fentanyl buccal soluble film, palliative care, proportional dose

## Abstract

**Background:**

The management of breakthrough pain (BTP) in cancer patients is a challenge. It is clinically useful to evaluate the effectiveness of rapid-onset opioid at a starting dose in proportional to the background opioid regimen. This open-label, multicenter, noncomparative study aimed to assess the efficacy and safety of proportional doses of fentanyl buccal soluble film (FBSF) in patients with breakthrough cancer pain.

**Methods:**

Thirty patients aged 20 to 70, experiencing 1 to 3 BTP per day, receiving regimens equivalent to 60 to 360 mg/day of oral morphine or 25 to 150 μg/h of transdermal fentanyl ≥1 week, were prospectively recruited. FBSF was administered proportionally based on their current opioid regimen for baseline pain. The percentage of patients requiring dose titration was evaluated. For each BTP episode, changes in pain intensity at 30 minutes (PID30) after dosing, patient's satisfaction, the percentage of episodes requiring rescue medication, and adverse events (AEs) were recorded.

**Results:**

The percentage of patients who required dose titration was 21.4% (6/28) and 12.0% (3/25) in the full analysis set and per-protocol populations, respectively. The average PID30 was 3.9, and a pain score ≤3 was achieved in 95.1% of the events. Eight out of 367 (2.2%) BTP episodes needed rescue medication. The majority of subjects (75.8%) rated their experience of pain management as good to excellent. A total of 6 drug-related AEs were reported by 3 (10.7%) patients in the safety population.

**Conclusions:**

FBSF dose in proportional to the regimen of opioid for baseline pain management is efficacious and well tolerated for the treatment of cancer patients with BTP.

## 1 Introduction

Breakthrough pain (BTP) is defined as a transitory exacerbation of pain experienced by cancer patients, who are currently under stable management for chronic pain.^[1,2]^ Despite receiving a regular dose of opioid, a sudden attack of relentless pain is an unquestionable burden for cancer patients and adversely affect their quality of life. Adequate analgesia should be achieved for cancer patients’ benefit.

With the characteristics of rapid onset and short duration, rapid-onset opioids (ROOs) are recommended for the treatment of BTP. Fentanyl buccal soluble film (FBSF; Onsolis, Breakyl, Painkyl) is one of the formulations of fentanyl, a potent synthetic opioid pain medication. Established using a new drug delivery system called BioErodible MucoAdhesive (BEMA), FBSF is comprised of 2 layers, a bioadhesive layer containing fentanyl citrate and an inactive layer that help prevent the active drug from diffusion.^[3]^ As a transmucosal form of ROOs, this formulation allows FBSF to be absorbed quickly and also avoids first-pass metabolism.

To appropriately and optimally manage BTP, a critical point is to quickly achieve an adequate dose for each cancer patient individually via an efficient way. To date, there is no literature providing related results in terms of strategies for establishing FBSF starting dosage in cancer patients suffering BTP. Administration of FBSF based on patients’ regimen for baseline pain control is a feasible approach in this regard.^[4]^ Thus, it would be of clinical significance to assess the efficacy of FBSF at a dose proportional to the around-the-clock (ATC) opioid regimen.

The aim of this study was to evaluate the effectiveness and safety of proportional doses of FBSF in patients with BTP. The primary objective was to evaluate the efficacy of proportional doses of FBSF by identifying the percentage of patients requiring dose titration. Changes in pain intensity (PI), subjects’ satisfaction, and percentage of episodes requiring rescue medication were analyzed as secondary endpoints. Adverse events (AEs) were recorded from a safety aspect. In addition, to provide further information, the relationship between ATC opioid dose and FBSF effective dose was also investigated. Herein, we report that proportional dose of FBSF based on patients’ ATC doses of analgesics provides an effective means for BTP management with good tolerance.

## 2 Methods

### 2.1 Study design

This was an open-label, multicenter, noncomparative study conducted at 3 clinical sites in Taiwan between January 2015 and June 2016. The trial was carried out in full accordance with the World Medical Association's Declaration of Helsinki and the Good Clinical Practice approved by the International Conference on Harmonization. The study protocol was approved by the institutional review board at each study site and written informed consent was obtained from all patients.

### 2.2 Patients

Subjects who were eligible to enter the trial should regularly experience 1 to 3 BTP episodes per day that required additional opioids for pain control. Subjects were receiving a stable regimen of opioids equivalent to 60 to 360 mg/day of oral morphine or 25 to 150 μg/h of transdermal fentanyl for 1 week or longer. At least partial relief of BTP was achieved by use of opioid therapy. All patients were between 20 and 70 years of age and were able to correctly self-administer study medication or had a caregiver to help correctly apply the study medication. In addition, subjects were willing and able to complete patient diary when pain episode occurred.

Patients who had rapidly escalating pain, histories of hypersensitivity or intolerance to fentanyl, or cardiopulmonary disease that would increase the risk of respiratory depression were excluded from the study. Patients with psychiatric/cognitive or neurological impairment that would limit their ability to understand or complete the diary were also excluded. Patients with moderate to severe mucositis or abnormal oral mucosa (that would impede drug absorption), with recent history or current evidence of alcohol or other drug substance abuse, use of an investigational drug within 4 weeks preceding the study, or patients who were pregnant, nursing, or had positive pregnancy test were not allowed to enter the study.

### 2.3 Study procedure

The study consisted a screening period, a treatment period, and a safety follow-up period. After screening, eligible subjects started with a proportional dose of FBSF (Painkyl/Onsolis; TTY Biopharm Company Limited/BioDelivery Sciences International, Inc) based on their ATC doses of analgesics (Table 1). If the ATC dose was between 2 dose levels listed in Table 1, the lower level dose was used for conversion. If adequate pain relief was not achieved, the patient may use a rescue medication at 30 minutes after dosing and titrated the dose of FBSF by 200 μg at each subsequent BTP episode until adequate pain relief was achieved. A certain dose of FBSF that achieved adequate pain relief with tolerable side effects for 2 consecutive BTP episodes was considered as an effective dose. The treatment of study medication was administered for a maximum period of 2 weeks (treatment period) unless lack of effect (cannot achieve adequate pain relief after administration of rescue medication), intolerable toxicity or consent withdrawal.

**Table 1:**
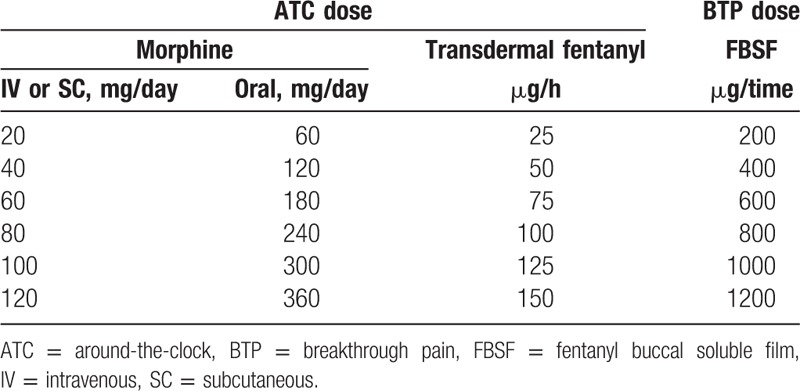
Dose of fentanyl buccal soluble film converted from current regimen of opioid.

### 2.4 Assessments

The efficacy of FBSF dose proportional to the baseline opioid regimen was evaluated by identifying the percentage of patients requiring dose titration. The secondary endpoints included the difference in pain intensity 30 minutes (PID30) after dosing, subjects’ satisfaction, and percentage of episodes requiring rescue medication.

PI was evaluated using an 11-point numeric scale with 0 = “no pain” to 10 = “worst pain.” For each BTP episode, PID30 was calculated by subtracting the pain score obtained 30 minutes after dosing from pain score obtained at baseline. The events of pain relief (pain scores ≤3 at 30 minutes after dosing) from severe (score 7–10) or moderate (score 4–6) pain were also counted and analyzed. A 5-point (poor, fair, good, very good, and excellent) categorical scale was used to assess the performance of study medication by questionnaire. At every episode, subjects recorded whether a rescue medication was taken after administration of study medication.

For safety measurement, the occurrence of AEs and serious adverse events (SAEs) was documented during and after study drug treatment. The severity and relationship of each AE and SAE were also recorded.

### 2.5 Statistical analysis

Full analysis set (FAS) contained subjects who received at least 1 dose of FBSF without major protocol violation. Per-protocol (PP) population included subjects who completed study treatment period. The safety population was defined as subjects who were exposed to at least 1 dose of FBSF and were available for follow-up safety information. All clinical efficacy outcomes were analyzed for the FAS and PP populations. Descriptive statistics were performed for all data collected. Although continuous variables were described with mean, standard deviation (SD), median, minimum and maximum, counts and percentages were calculated for categorical data. The correlation coefficient between ATC dose and effective dose was analyzed using Spearman rank correlation. A *P* value of <.05 was considered to indicate statistical significance, and all tests were 2 tailed. All analyses and summaries were produced using SAS software (SAS Institute Inc, Cary, NC) Version 9.4.

## 3 Results

### 3.1 Patient disposition and demographics

**Figure 1:**
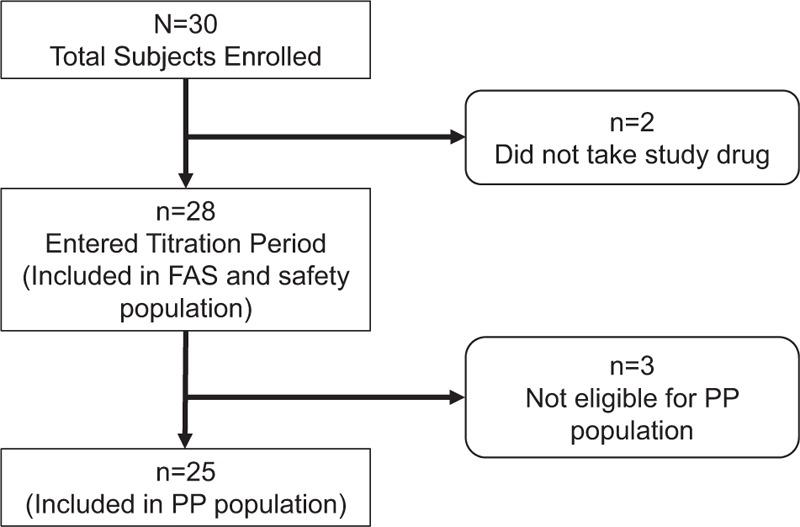
Patient disposition. FAS = full analysis set, PP = per-protocol.

A total of 30 subjects were screened and enrolled into the study. Two subjects did not receive any study medication, and 28 subjects who took at least 1 dose of FBSF were included in the FAS and safety populations. Of the 28 subjects, 3 subjects were excluded, and 25 subjects were included in the PP population (Fig. 1).

Table 2 summarizes the patients’ demographic characteristic. The FAS population had a mean age of 53.3 years, with 15 (53.6%) male subjects. In the FAS population, the median ATC dose (equivalent oral morphine) was 75 mg/day, ranged from 60 to 360 mg/day. In the PP population, the median ATC dose was 90 mg/day, with a range of 60 to 360 mg/day. A majority of BTP episodes (79.6%; 305/383 events) was rated as moderate (4–6) in PI, whereas 20.4% (78/383 events) of the events were rated as severe (7–10).

**Table 2:**
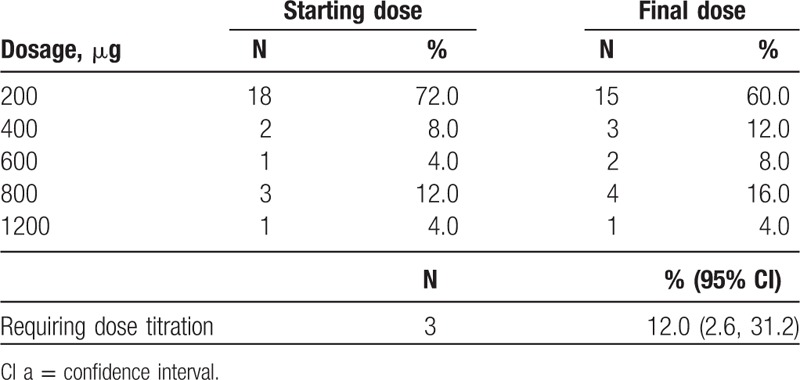
Patient characteristics (full analysis set).

### 3.2 Efficacy

**Figure 2:**
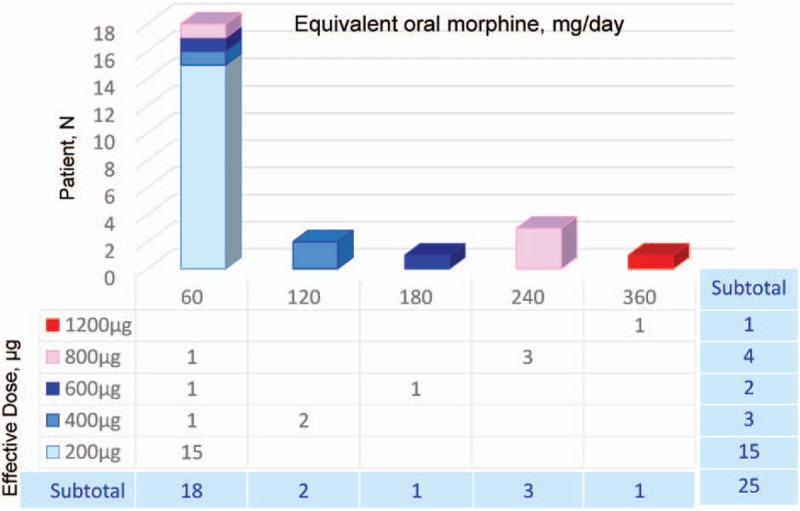
The correlation between around-the-clock (ATC) dose and effective dose of fentanyl buccal soluble film [FBSF; the per-protocol (PP) population, N = 25] was displayed. A total of 25 patients completed the treatment period were included in the PP population. For each patient, the ATC dose of oral morphine (mg/day) and its corresponding effective FBSF dose (μg) are presented. A positive correlation was identified between the ATC dose and the effective FBSF dose (Spearman rank correlation; *r*_*s*_ = 0.80; *P* < .0001).

In the PP populations, 60%, 12%, 8%, 16%, and 4% of the patients achieved a final effective FBSF dose of 200, 400, 600, 800, and 1200 μg, respectively (Table 3). There were 3 subjects [12.0%; 95% confidence interval (CI): 2.6, 31.2] in the PP population who required dose titration. The correlation between ATC dose and effective dose is illustrated in Figure 2, and a positive correlation was identified (Spearman rank correlation; *r*_s_ = 0.80; *P* < .0001).

**Table 3:**
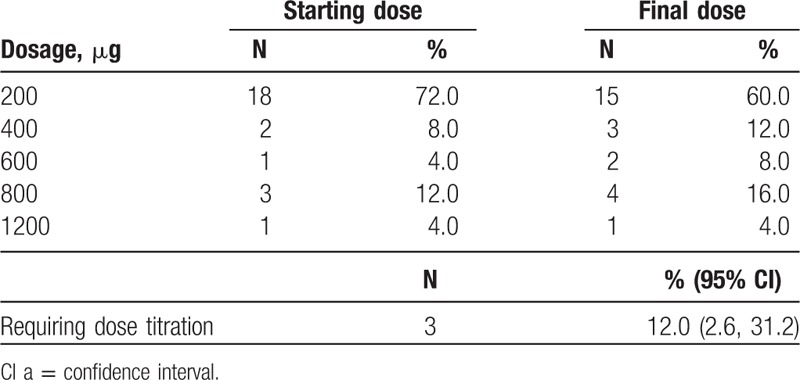
Effective dose of fentanyl buccal soluble film in the per-protocol population.

**Figure 3:**
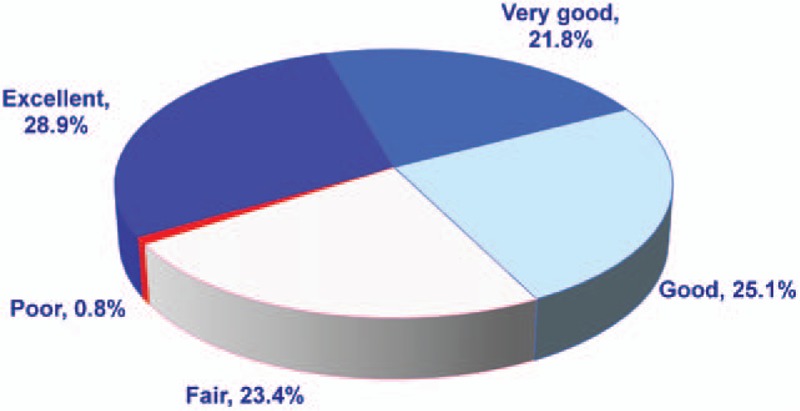
Overall satisfaction in the per-protocol (PP) population [N = 25, breakthrough pain (BTP) = 367] was shown. The pie chart represents the patients’ satisfaction at 30 minutes after taking fentanyl buccal soluble film (FBSF). A total of 367 episodes were recorded in the PP population. Of the 367 episodes recorded, 75.8% of the events were rated as good to excellent.

In the PP population, a total of 367 BTP events were recorded during the treatment period. At 30 minutes after FBSF administration, a decrease in PI was observed, with a mean PID30 of 3.9 (Table 4). A pain relief (pain scores ≤3) from severe and moderate pain was achieved in 81.6% and 98.6% of the events, respectively. Only 8 out of 367 BTP episodes required rescue medication (2.2%; 95% CI: 1.0, 4.3). Of the 367 episodes recorded, 75.8% of the events were rated as good or above (Fig. 3).

**Table 4:**
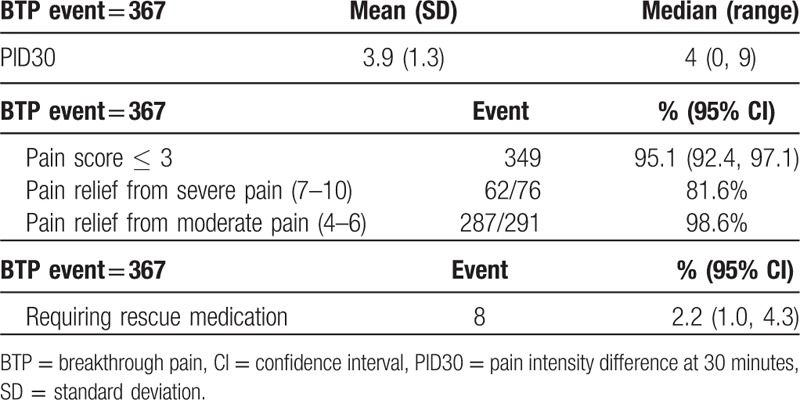
Analysis of pain intensity difference at 30 minutes after dosing in the per-protocol population (breakthrough pain events = 367).

### 3.3 Overall safety

A total of 46 AEs was recorded in the safety population. Overall, 40 (87.0%) AEs recorded in 20 patients were not considered to be drug related. There were 6 drug-related AEs reported by 3 subjects (10.7% of the safety population), including skin itching (3.6%), nausea (3.6%), dizziness (7.2%), vomiting (3.6%), and anorexia (3.6%). All AEs were of grade 1 to 2 in severity (Table 5). Seven SAEs were reported by 7 patients. None of these events were considered to be drug related.

**Table 5:**
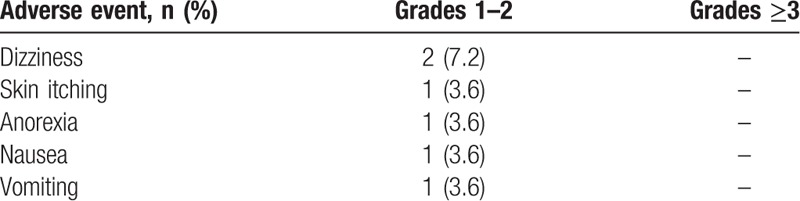
Incidence of drug-related adverse events in the full analysis set.

## 4 Discussion

This was an open-label, noncomparative study aimed to assess the efficacy and safety of FBSF dosage in proportional to the background opioid regimen. Dose proportional to the basal opioid regimen has been investigated in fentanyl buccal tablet (FBT)^[5,6]^ but not FBSF. In fact, only a limited number of studies that associated with FBSF are currently available. To our knowledge, there is no study focusing on FBSF dose proportional to ATC regimen, and no literature specifically focusing on Asian in particular. In addition to the low interindividual variability (coefficients of variation 7%–11%) of FBSF.^[7]^ Fentanyl maximum plasma concentration (*C*_max_) increased in a linear manner after administration of FBSF doses of 200–1200 μg.^[8]^ Moreover, in a randomized controlled study comparing dose titration with proportional doses, the number of episodes requiring rescue medication was significantly higher in patients underwent dose titration of FBT for the first episode of BTP.^[9]^ These favorable features of FBSF and the advantage of proportional dose prompt us to further explore the proportionality between the BTP dose of FBSF and baseline opioid regimen.

The design of the conversion table (Table 1) is based on experiences in clinical practices, as an oral BTP medication is generally given in doses of 1/6 of the ATC dose.^[10]^ Although the absolute availability of fentanyl from FBSF is approximately 71%, approximately 51% of the administered FBSF is immediately absorbed through the buccal mucosa, and the remaining 20% is absorbed later from the gastrointestinal tract.^[11]^ Thus, approximately 100 μg fentanyl is immediately absorbed from a 200-μg FBSF, which corresponds to 1/6 of a transdermal fentanyl dose of 25 μg/h (i.e., 600 μg/day). In addition, a maximum of 4 times a day is allowed for FBSF application. For a patient with an effective dose of 200 μg, the maximum FBSF dose is 800 μg. Since the absolute availability of fentanyl is 71%, approximately 568 μg of fentanyl enters one's systematic circulation eventually, which equals to 23.7 μg/h FBSF, a dose close to corresponding 25 μg/h in the conversion table.

In the present study, the effectiveness of the FBSF starting dose was evaluated as the percentage of patients requiring dose titration. In the PP population, only 3 subjects (12.0%; 95% CI: 2.6, 31.2) required further dose titration, all of whom started at a dose of 200 μg and were identified with a final dose of 400, 600, and 800 μg FBSF. Notably, the majority (80.0%) of the subjects received ATC doses equivalent to 60 to 120 mg/day of oral morphine, which is because that patients with high ATC doses tended to be excluded from the study due to physical conditions and disease progression. Thus, in the present study, 72% of the subjects were found with FBSF final doses of 200 or 400 μg.

In the management of BTP, the strategy of proportional dose possesses several advantages in comparison with traditional dose titration. Using dose proportional to background opioid regimen shortens the time needed for identifying an effective dose. Although dose titration is apparently a safe way to determine an effective FBSF dose, it is time consuming and potentially reduces patients’ compliance. As effective doses were directly identified in 88% of the subjects without the need of further titration, it indicates that the FBSF starting dose converted from regimens used in chronic pain therapy provides a practical approach for FBSF administration.

The analyses of secondary endpoints (difference in PI, patients’ satisfaction, and episodes required rescue medication) further support the feasibility of starting FBSF treatment dose in proportional to chronic pain therapy. It was observed that the mean PID30 was 3.9, with an SD of 1.3. Nevertheless, a severe pain episode (PI rated 7–10) being relieved is different from a PI reduced from moderate (PI rated 4–6) to mild (PI 0–3). In our results, a pain relief (defined as a PI score ≤3 after treatment) from a severe and moderate episode was achieved in 81.6% and 98.6% of the events, respectively. Thus, it suggested that FBSF provides effective pain relief from both severe and moderate pain.

In the PP population, only 2.2% (8/367) of the BTP episodes required rescue medication during the treatment period. Also, approximately 75.8% of the events were rated as good to excellent, and only 0.8% of the events were rated as poor. Notably, although around one-fourth of the events were rated as fair, the majority of these episodes (87.2%; 75/86 events) displayed a postmedication pain score ≤3, indicating the pain was effectively relieved in these subjects. The results mentioned above reflect the efficacy of a proportional dose of FBSF. However, limited by the study design, only patients under background pain treatment for at least 1 week were recruited, and the ATC dose could not be adjusted after entry into the treatment period. Hence, the disease progression itself may affect and underestimate the results of efficacy evaluation.

During the study period, the majority (87%) of AEs and all SAEs recorded were not drug related. Only a total of 6 drug-related AEs were reported by 3 subjects (10.7%) with grades 1 to 2 in severity. It suggests that the dose of FBSF proportional to daily opioid regimen provides a tolerable and efficient means of administration without concerns of overdosing. Nevertheless, the risk of unintentional overdose should be kept in mind. Like other Schedule II controlled substances, drugs that have a high potential for abuse, the risks of overdose, misuse, and diversion are associated with fentanyl.^[12–14]^ In a long-term (18-month) study of FBT, among the 646 patients in the maintenance safety analysis set, 4 patients experienced AEs of abuse or drug dependence and 2 events were FBT related.^[15]^ In another long-term study of fentanyl pectin nasal spray (FPNS), 1 event of unintentional FPNS overdose occurred during the treatment of a severe BTP episode, 1 case of possible diversion was reported, and no cases of drug abuse were recorded.^[16]^ These studies showed a low incidence of overdose, abuse, and diversion. Unintentional overdoses could, however, be driven by the need for increased efficacy after severe BTP episode. With that being said, proper management of medication is required. The occurrence of overdose could be reduced through enhancing patient education and the collaboration between prescriber and educational professionals/pharmacists.

In the present study, one of the exclusion criteria was patients with moderate to severe mucositis. In fact, all patients enrolled had no medical histories or commodities of mucositis. In an open-label, single-dose study using 200 μg FBSF, opioid-naïve patients with grade I mucositis were associated with decreased fentanyl exposure as compared with matched controls.^[17]^ No application site irritation was reported by study subjects. The study suggests that application of FBSF to an area of grade I mucositis does not cause increased fentanyl exposure or oral mucosal irritation. Although the *C*_max_ values were lower in the oral mucositis cohort, it suggested that the difference is not clinically relevant, and dose adjustment is not required.

Previous literature of FBTs and oral transmucosal fentanyl citrate suggest that there was no correlation between the effective dose of BTP medication and background opioid dose (fixed schedule dose).^[18–20]^ Opposite to the aforementioned studies, a study conducted by Hagen et al^[21]^ using pooled data from 3 trials showed a statistically significant relationship between BTP dose and ATC dose. In our study, a positive correlation between the FBSF effective dose and the ATC dose was found. Although few subjects were under high-dose ATC and FBSF treatment, equianalgesic ratios provided in this study offer a reference estimate for physicians to convert ATC opioid dose to short-acting FBSF dose, which is always a challenge for transmucosal immediate-released fentanyl.^[22]^

Collectively, with the high prevalence and pervasive impact on cancer patients, BTP should be well-managed. As an immediate-release, rapid-onset form of fentanyl, FBSF serves as a new option for physicians in ameliorating patients’ quality of life. The results of the present study demonstrated that FBSF dose in proportional to the regimen of opioid for baseline pain management was efficacious and was well tolerated for the treatment of cancer patients suffering BTP. This was the first study investigating the feasibility of proportional dose for FBSF. For the reasons that BTP presents varied characteristics among patients with different types of cancer, future studies may be conducted to explore the efficacy and safety profiles for patients with certain categories of cancer. A long-term study of FBSF with larger subject number may be needed as well for further safety evaluations.

## Author contributions

**Conceptualization:** Tsung-Yu Yen, Jeng-Fong Chiou, Wei-Yong Chiang, Wen-Hao Su, Shen-Chi Wu, Yuen-Liang Lai.

**Data curation:** Tsung-Yu Yen, Jeng-Fong Chiou, Wei-Yong Chiang, Wen-Hao Su, Ming-Yuan Huang, Ming-Hung Hu, Shen-Chi Wu, Yuen-Liang Lai.

**Formal analysis:** Tsung-Yu Yen, Shen-Chi Wu, Yuen-Liang Lai.

**Investigation:** Tsung-Yu Yen, Jeng-Fong Chiou, Wei-Yong Chiang, Wen-Hao Su, Ming-Yuan Huang, Ming-Hung Hu, Shen-Chi Wu, Yuen-Liang Lai.

**Methodology:** Tsung-Yu Yen, Jeng-Fong Chiou, Wei-Yong Chiang, Wen-Hao Su, Ming-Yuan Huang, Ming-Hung Hu, Shen-Chi Wu, Yuen-Liang Lai.

**Project administration:** Tsung-Yu Yen, Jeng-Fong Chiou, Wei-Yong Chiang, Wen-Hao Su, Ming-Yuan Huang, Shen-Chi Wu, Yuen-Liang Lai.

**Resources:** Tsung-Yu Yen, Jeng-Fong Chiou, Wei-Yong Chiang, Wen-Hao Su, Shen-Chi Wu, Yuen-Liang Lai.

**Software:** Tsung-Yu Yen, Shen-Chi Wu, Yuen-Liang Lai.

**Supervision:** Tsung-Yu Yen, Jeng-Fong Chiou, Wei-Yong Chiang, Wen-Hao Su, Shen-Chi Wu, Yuen-Liang Lai.

**Validation:** Tsung-Yu Yen, Jeng-Fong Chiou, Wei-Yong Chiang, Wen-Hao Su, Ming-Yuan Huang, Ming-Hung Hu, Shen-Chi Wu, Yuen-Liang Lai.

**Visualization:** Tsung-Yu Yen, Ming-Yuan Huang, Shen-Chi Wu, Yuen-Liang Lai.

**Writing – original draft:** Tsung-Yu Yen, Jeng-Fong Chiou, Wei-Yong Chiang, Wen-Hao Su, Ming-Yuan Huang, Ming-Hung Hu, Shen-Chi Wu, Yuen-Liang Lai.

**Writing – review and editing:** Tsung-Yu Yen, Jeng-Fong Chiou, Shen-Chi Wu, Yuen-Liang Lai.

## References

[1] Mercadante S Breakthrough pain in cancer patients: prevalence, mechanisms and treatment options. Curr Opin Anaesthesiol 2015;28:559–64. 26263120 10.1097/ACO.0000000000000224

[2] Portenoy RK Hagen NA Breakthrough pain: definition, prevalence and characteristics. Pain 1990;41:273–81. 1697056 10.1016/0304-3959(90)90004-W

[3] Garnock-Jones KP Fentanyl buccal soluble film: a review in breakthrough cancer pain. Clin Drug Investig 2016;36:413–9. 10.1007/s40261-016-0394-y 27007271

[4] Mercadante S Villari P Ferrera P Transmucosal fentanyl vs intravenous morphine in doses proportional to basal opioid regimen for episodic-breakthrough pain. Br J Cancer 2007;96:1828–33. 17519902 10.1038/sj.bjc.6603811 PMC2359971

[5] Mercadante S Porzio G Aielli F The use of fentanyl buccal tablets for breakthrough pain by using doses proportional to opioid basal regimen in a home care setting. Support Care Cancer 2013;21:2335–9. 23564072 10.1007/s00520-013-1799-2

[6] Kosugi T Hamada S Takigawa C A randomized, double-blind, placebo-controlled study of fentanyl buccal tablets for breakthrough pain: efficacy and safety in Japanese cancer patients. J Pain Symptom Manage 2014;47:990–1000. 24099893 10.1016/j.jpainsymman.2013.07.006

[7] Davies A Finn A Tagarro I Intra- and interindividual variabilities in the pharmacokinetics of fentanyl buccal soluble film in healthy subjects: a cross-study analysis. Clin Drug Investig 2011;31:317–24. 10.1007/BF03256930 21294598

[8] Finn AL Vasisht N Stark JG Dose proportionality and pharmacokinetics of fentanyl buccal soluble film in healthy subjects: a phase I, open-label, three-period, crossover study. Clin Drug Investig 2012;32:63–71. 10.2165/11594670-000000000-00000 22128878

[9] Mercadante S Gatti A Porzio G Dosing fentanyl buccal tablet for breakthrough cancer pain: dose titration versus proportional doses. Curr Med Res Opin 2012;28:963–8. 22480130 10.1185/03007995.2012.683112

[10] Hanks GW Conno F Cherny N Morphine and alternative opioids in cancer pain: the EAPC recommendations. Br J Cancer 2001;84:587–93. 11237376 10.1054/bjoc.2001.1680 PMC2363790

[11] ONSOLIS [package insert]. Raleigh, NC: IBSI Inc; 2016.

[12] Fine PG Narayana A Passik SD Treatment of breakthrough pain with fentanyl buccal tablet in opioid-tolerant patients with chronic pain: appropriate patient selection and management. Pain Med 2010;11:1024–36. 20642730 10.1111/j.1526-4637.2010.00891.x

[13] McDonald J Opioid prescribing: guidelines, laws, rules, regulations, policies, best practices. R I Med J (2013) 2013;96:38–41. 24187678

[14] Fentanyl buccal soluble film (Onsolis) for breakthrough cancer pain. Med Lett Drugs Ther 2010;52:30–1. 20407416

[15] Fine PG Messina J Xie F Long-term safety and tolerability of fentanyl buccal tablet for the treatment of breakthrough pain in opioid-tolerant patients with chronic pain: an 18-month study. J Pain Symptom Manage 2010;40:747–60. 20594801 10.1016/j.jpainsymman.2010.02.009

[16] Taylor D Radbruch L Revnic J A report on the long-term use of fentanyl pectin nasal spray in patients with recurrent breakthrough pain. J Pain Symptom Manage 2014;47:1001–7. 24128821 10.1016/j.jpainsymman.2013.07.012

[17] Finn AL Hill WC Tagarro I Absorption and tolerability of fentanyl buccal soluble film (FBSF) in patients with cancer in the presence of oral mucositis. J Pain Res 2011;4:245–51. 21941456 10.2147/JPR.S22641 PMC3176141

[18] Coluzzi PH Schwartzberg L Conroy JD Breakthrough cancer pain: a randomized trial comparing oral transmucosal fentanyl citrate (OTFC) and morphine sulfate immediate release (MSIR). Pain 2001;91:123–30. 11240084 10.1016/s0304-3959(00)00427-9

[19] Davies AN Dickman A Reid C The management of cancer-related breakthrough pain: recommendations of a task group of the Science Committee of the Association for Palliative Medicine of Great Britain and Ireland. Eur J Pain 2009;13:331–8. 18707904 10.1016/j.ejpain.2008.06.014

[20] Portenoy RK Taylor D Messina J A randomized, placebo-controlled study of fentanyl buccal tablet for breakthrough pain in opioid-treated patients with cancer. Clin J Pain 2006;22:805–11. 17057563 10.1097/01.ajp.0000210932.27945.4a

[21] Hagen NA Fisher K Victorino C A titration strategy is needed to manage breakthrough cancer pain effectively: observations from data pooled from three clinical trials. J Palliat Med 2007;10:47–55. 17298253 10.1089/jpm.2006.0151

[22] Chang A Roeland EJ Atayee RS Transmucosal immediate-release fentanyl for breakthrough cancer pain: opportunities and challenges for use in palliative care. J Pain Palliat Care Pharmacother 2015;29:247–60. 26368648 10.3109/15360288.2015.1063560

